# The Role of Monoclonal Antibodies in the First-Line Treatment of Transplant-Ineligible Patients with Newly Diagnosed Multiple Myeloma

**DOI:** 10.3390/ph14010020

**Published:** 2020-12-29

**Authors:** Francesca Bonello, Mariella Grasso, Mattia D’Agostino, Ivana Celeghini, Alessia Castellino, Mario Boccadoro, Sara Bringhen

**Affiliations:** 1 Myeloma Unit, Division of Hematology, University of Torino, Azienda Ospedaliero-Universitaria Città della Salute e della Scienza di Torino, 10126 Torino, Italy; 2 S.C. Ematologia, Azienda Ospedaliera Santa Croce-Carle, 88900 Cuneo, Italy

**Keywords:** multiple myeloma, monoclonal antibodies, transplant-ineligible patients, new diagnosis, first-line treatment

## Abstract

Elderly transplant-ineligible (NTE) patients represent the majority of patients affected by multiple myeloma (MM). Elderly patients are a highly heterogeneous population, with large variability in health and functional status. Thus, choosing their optimal treatment is challenging. A wide range of first-line treatments is available, and novel-agent combinations, including monoclonal antibodies (mAbs), have recently entered clinical practice. The combination of the anti-CD38 mAb daratumumab with bortezomib, melphalan and prednisone (Dara-VMP) or lenalidomide and dexamethasone (Dara-Rd) demonstrated impressive advantages in terms of progression-free survival and minimal residual disease negativity, as compared to VMP and Rd, without safety concerns. Another anti-CD38 mAb, isatuximab, is showing encouraging results, and new isatuximab-based combinations might enter clinical practice in the future. Nevertheless, available data come from clinical trials with selected patient populations and, to date, the manageability of these regimens in real-life patients or in frail patients remains unknown. Frailty-tailored treatments, including mAbs, are under evaluation in preliminary studies. In this review, we analyze recently approved mAb-based treatments for NTE newly diagnosed MM patients and new combinations under evaluation, focusing on the efficacy and safety of these regimens and on open issues regarding the choice of therapy for elderly patients.

## 1. Introduction

Multiple myeloma (MM) is the second most common hematologic malignancy, with an incidence of approximatively 5 new cases per 100,000 people/year in Western countries [1]. MM predominantly affects elderly patients, with a median age at diagnosis of 69 years and about 30% of new diagnoses occurring in patients older than 75 years of age [2].

Traditionally, patients aged >65 years were considered not suitable for high-dose therapies and autologous stem-cell transplantation (ASCT). Currently, the cut-off age for ASCT eligibility has been reconsidered and raised to 70 years in the real-life setting, as well as in most clinical trials [3]. Some centers perform ASCT (with or without reduced-intensity conditioning) in selected patients up to 75 years of age in good physical conditions, with good tolerability and low transplant mortality rate (1–2%) [4,5]. In the last years, a steady survival improvement has been observed in MM patients treated with novel agents plus ASCT [6]. On the other hand, evidence about an increased overall survival (OS) in transplant-ineligible (NTE) patients was more controversial [7,8,9]. Recent data reported a significant OS improvement from 2004 to 2017 also for patients aged 65–75 years (4-year OS estimates from 48% to 75%) and ≥75 years (from 24% to 56%) [10].

The advent of immunotherapy revolutionized the treatment scenario for MM patients [11,12,13,14]. The anti-CD38 monoclonal antibody (mAb) daratumumab has been included in the standard-of-care regimens for NTE newly diagnosed (ND)MM patients following the results of the phase III ALCYONE trial, which compared daratumumab plus bortezomib, melphalan and prednisone (Dara-VMP) to VMP, and the MAIA trial, which compared daratumumab plus lenalidomide and dexamethasone (Dara-Rd) to Rd [15,16]. Moreover, other agents such as the anti-CD38 mAb isatuximab and the anti-SLAMF7 elotuzumab have already been approved at relapse and are under evaluation in the first-line setting.

Despite the growing range of treatment options, elderly NTE patients represent a heterogeneous population, including fit patients who are able to tolerate full-dose triplets/quadruplets and frail patients requiring tailored approaches with less toxic combinations [17,18,19]. New mAb-based treatment combinations are being explored also in these subsets of patients.

This review provides an overview about front-line treatments including mAbs for NTE MM patients, focusing on recently approved daratumumab-containing regimens and on new combinations under clinical evaluation.

## 2. How to Choose Therapy in NTE NDMM Patients

Elderly NTE patients represent a highly heterogeneous population, with large variability in chronological age, comorbidities and functional status. Consequently, not all patients can receive the same treatment, and intensity and therapeutic goals should be modulated according to patient fitness. The detection of frailty (defined as a state of increased vulnerability and decreased ability to adapt to a stressor) is of the outmost importance for the selection of the appropriate treatment [20,21].

Several tools have been proposed to detect frailty. To date, the gold standard is the International Myeloma Working Group (IMWG) frailty score [17,22], which classifies patients as fit, intermediate-fit and frail according to age, comorbidities assessed through the Charlson Comorbidity Index (CCI), and functional status assessed through patient reported questionnaires (Katz Index of Independence in Activities of Daily Living and Lawton Instrumental Activities of Daily Living). It was developed through the analysis of more than 800 NDMM patients treated with novel agents (lenalidomide, bortezomib, or carfilzomib) in clinical trials. This score was found to be predictive of an inferior OS in intermediate-fit (hazard ratio [HR] 1.61, *p* = 0.04) and frail patients (HR 3.57, *p* < 0.01), as compared to fit patients, and of a higher risk of non-hematologic toxicity and treatment discontinuation [23].

Other models have been proposed, aiming at increasing the discriminative power in the detection of frailty and at simplifying the score. Among the most recent ones, Facon et al. proposed a simplified frailty score including age, CCI, and performance status to discriminate between fit and frail patients, the latter with a higher risk of death (HR 1.86, *p* < 0.01) and treatment discontinuation (HR 1.66, *p* < 0.01) [24]. Cook et al. developed a score including performance status, age, International Staging System (ISS) stage, and circulating levels of C-reactive protein to identify NTE patients at low, intermediate, and high risk for OS and early mortality [25].

**Figure 1:**
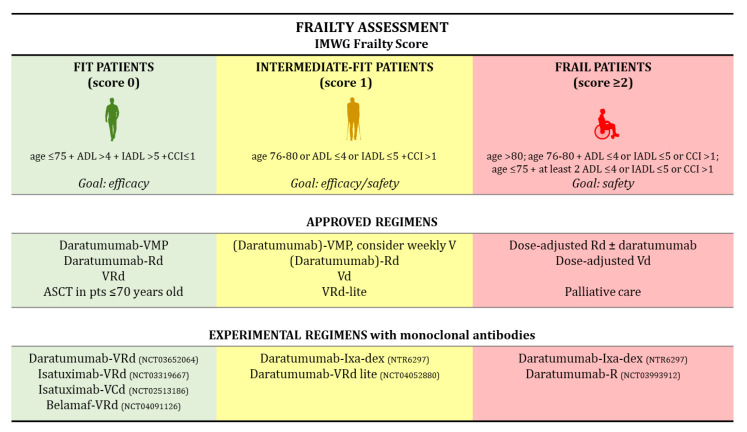
Frailty Assessment. Abbreviations: IMWG, International Myeloma Working Group; ADL, Activities of Daily Living; IADL, Instrumental Activities of Daily Living; CCI, Charlson Comorbidity Index; V, bortezomib; Vd, bortezomib, dexamethasone; VMP, bortezomib, melphalan, prednisone; Rd, lenalidomide, dexamethasone; VRd, bortezomib, lenalidomide, dexamethasone; VRd-lite, VRd with dose-attenuated subcutaneous bortezomib (V); ASCT, autologous stem-cell transplantation; VCd, bortezomib, cyclophosphamide, dexamethasone; Ixa, ixazomib; Belamaf, belantamab mafodotin; pts, patients.

Fit patients should receive full-dose triplets/quadruplets including novel agents. ASCT can be considered in selected cases with good organ function [17] (Figure 1).

In these patients, the treatment goal is obtaining disease remission and deep responses with minimal residual disease (MRD) negativity. Fit patients represent the vast majority of patients enrolled in phase III clinical trials, since those with significant comorbidities or poor performance status are usually excluded. Moreover, patients <75 years of age represent 60–70% of the study population enrolled in phase III clinical trials [15,16]. In these patients, the combination of daratumumab with VMP or Rd demonstrated efficacy, despite a slightly higher rate of toxicity than VMP and Rd. Intermediate-fit patients are candidates for dose-adapted triplets or doublets balancing efficacy and safety, whereas frail patients should receive low-intensity regimens aimed at preserving quality of life and reducing the burden of MM-related symptoms [17]. Initial data on the benefit of a treatment schema in frailty-defined subgroups of patients are beginning to emerge [26,27].

To date, whether new mAb-based regimens could be safely administered to intermediate-fit and frail patients remains unanswered. Indeed, neither frailty assessment nor analysis of outcome according to frailty status are yet available for these trials. Nevertheless, intermediate-fit and frail patients represent a large proportion of patients in everyday clinical practice. Real-life data about patients treated with mAbs in the first line of treatment are not yet available but will be particularly useful to shed light on this topic. Remarkably, new phase II trials are exploring frailty-tailored approaches including mAbs in less toxic combinations in NDMM patients. For instance, the HOVON group is evaluating the combination of daratumumab, ixazomib, and low-dose dexamethasone in intermediate-fit and frail patients [28]. The IFM2017_03 study will evaluate subcutaneous daratumumab plus lenalidomide vs. standard Rd in frail patients as a dexamethasone-sparing strategy, since steroids are scarcely tolerated in the long term, particularly in elderly populations (NCT03993912).

## 3. Monoclonal Antibodies in Elderly Patients: Data about Feasibility

In this section, we will address the feasibility of mAbs added to standard-of-care, backbone combinations in elderly patients. The majority of available evidence comes from subgroup analyses according to age in large randomized phase III trials that evaluated combinations with or without mAbs.

### 3.1. Data on Relapsed/Refractory (RR)MM Patients

In RRMM patients, the POLLUX and CASTOR trials evaluated the addition of daratumumab to the backbones Rd and bortezomib-dexamethasone (Vd), respectively [29]. The efficacy and safety of daratumumab were tested in two different age groups: 65–74 years and ≥75 years [29]. In both trials, about 11–12% of patients were ≥75 years, and 37–41% of patients were 65–74 years old. In both age groups, efficacy in the daratumumab arm vs. the control arm was comparable both in terms of progression-free survival (PFS, POLLUX: HR 0.27 in age ≥75, HR 0.40 in age 65–74; CASTOR: HR 0.26 in age ≥75, HR 0.25 in age 65–74) and overall response rate (ORR, POLLUX: ORR 93% vs. 77% in age ≥75, ORR 93% vs. 80% in age 65–74; CASTOR: ORR 95% vs. 79% in age ≥75, ORR 83% vs. 62% in age 65–74). Regarding safety, infusion-related reactions (IRRs) occurred in similar rates in both age groups in the experimental arms of the two trials. IRRs were usually mild (grade [G]1–2) and did not result in treatment discontinuation. In the POLLUX trial, neutropenia was the most common hematologic G3–4 adverse event (AE), and pneumonia the most common non-hematologic G3–4 AE, although no increased toxicity was observed in patients aged ≥75 years. Lenalidomide dose intensity was lower in patients aged ≥75 years than in patients aged 65 to 74 years. However, this was not related to the use of a mAb, since it was observed in both the daratumumab arm and the control arm. In the CASTOR trial, thrombocytopenia was the most common hematologic G3–4 AE, while pneumonia and peripheral neuropathy were the most common non-hematologic G3–4 AEs. No clear signs of increased toxicity were observed in patients aged ≥75 years in the daratumumab arm.

The phase III ICARIA-MM trial evaluated the addition of the anti-CD38 mAb isatuximab to pomalidomide-dexamethasone (Pd) in heavily pretreated MM patients [30]. Among 307 total patients, 61 were aged ≥75 years (20%), 122 65–74 years (40%), and 124 <65 years (40%). In all three age groups, isatuximab-Pd significantly prolonged PFS compared to Pd, with a similar HR (0.45 in ≥75 years, 0.64 in 65–74 years, 0.64 in <65 years). The ORR was consistent with that observed in the overall population, with an advantage of isatuximab-Pd vs. Pd (ORR 53–64% vs. 31–39% across the three age subgroups). Health-related quality of life (QoL) was improved by the addition of isatuximab to Pd; this was also observed in patients aged ≥75 years. The preservation of QoL in this age group is important because it is often associated with the absence of MM-related and therapy-related complications that tend to be severe and debilitating in older patients. Regarding safety, IRRs were commonly observed in the isatuximab arm, although, interestingly, a trend towards a lower rate of IRRs was observed in patients aged ≥75 years (28.1%), as compared with patients aged 65–74 years (36.4%) or <65 years (42.6%). Although the biological mechanisms are unknown, the senescence of the immune system that is less prone to produce overreactions might play a role in this finding. The most common G ≥ 3 non-hematologic AE was pneumonia. In the isatuximab arm, its incidence was lower in elderly patients, but this may be due to a higher percentage of older patients receiving prophylactic antibiotics. An increased rate of acute kidney injury was found in patients aged ≥75 years vs. <65 years, especially in the isatuximab arm. This is possibly due to a higher tumoricidal effect of the 3-drug combination and to the lower renal buffer in elderly patients.

### 3.2. Data on NDMM Patients 

In NTE NDMM patients, a subanalysis according to age (≥75 years vs. <75 years) in the ALCYONE trial evaluating daratumumab-VMP vs. VMP was performed [31]. About 30% of the total trial population was ≥75 years old, and the efficacy benefit of daratumumab-VMP over VMP was confirmed (HR 0.53) and was similar to that observed in patients aged <75 years (HR 0.49). In both age groups, the ORRs (88% vs. 70% in ≥75 years; 92% vs. 76% in <75 years) and the rates of MRD negativity (24% vs. 8% in ≥75 years; 22% vs. 6% in <75 years) were higher in the daratumumab arm vs. the control arm, respectively. No safety issues were observed in patients aged ≥75 years, as compared to the overall population. Similarly, a subanalysis according to patient age was performed in the MAIA trial (Dara-Rd vs. Rd). Again, the PFS benefit observed in the Dara-Rd arm was also maintained in >75-year-old patients (HR 0.53 in <75 years old vs. 0.63 in ≥75 years old). As in the overall population, the main G ≥ 3 AEs in patients aged ≥75 years receiving Dara-Rd were neutropenia (60%) and pneumonia (15%), without emerging safety concerns, although lenalidomide dose intensity was slightly inferior in older patients (79% vs. 66%) [32]. Similar data on safety and efficacy confirmed the feasibility of the addition of daratumumab to Rd and VMP also in elderly East Asian patients [33,34]. The feasibility of the addition of a mAb to bortezomib-lenalidomide-dexamethasone (VRd) in NTE NDMM patients is currently being investigated in the randomized phase III trials CEPHEUS (daratumumab-VRd vs. VRd) and IMROZ (isatuximab-VRd vs. VRd).

The anti-SLAMF7 mAb elotuzumab is a very well tolerated drug that can be added to backbone treatments without significant toxicity [35]. Moreover, a subgroup analysis demonstrated similar efficacy across different age and ethnicity groups [36,37].

Together, these data suggest that mAbs can be added to backbone treatments even in elderly patients, due to their good safety profile. However, age-defined subgroup analyses are not robust enough to draw definitive conclusions. A sub-analysis according to frailty status should be conducted in order to confirm the aforementioned data. Moreover, clinical trial data should be confirmed in the real-world setting, where frail and elderly patients are more represented.

## 4. Clinical Trials Evaluating MAb-Based Treatments in NTE MM Patients 

Data about efficacy and safety in the main clinical trials evaluating mAb-based frontline therapy in NTE NDMM patients are summarized in Table 1.

### 4.1. New Standards of Care for NTE Patients

Data have been recently reported from two large phase III, multicenter, international, randomized trials evaluating daratumumab-based treatment combinations in NTE NDMM patients [16,38].

The ALCYONE trial [38] was a multicenter, randomized, open-label, active-controlled, phase III study that included previously untreated MM patients who were determined to be NTE because of their age (>65 years old) or substantial comorbidities. Patients were randomized 1:1 to receive standard VMP treatment (standard arm) vs. Dara-VMP (experimental arm). In the Dara-VMP group, intravenous daratumumab was added to the standard VMP schedule at the dose of 16 mg/kg of body weight, once weekly during cycle 1, once every 3 weeks in cycles 2–9, and once every 4 weeks thereafter as maintenance therapy until disease progression or unacceptable toxicity.

A total of 706 patients were enrolled: 350 in the Dara-VMP group and 356 in the VMP group. ORR, very good partial response (VGPR), and complete response (CR) rates were significantly higher in the Dara-VMP arm than in the VMP arm (Table 1).

The rate of MRD negativity (measured by next generation sequencing [NGS] at a threshold of 1 tumor cell per 105 white cells) was 4 times higher in the Dara-VMP group than in the VMP group (28% vs. 7%, respectively, *p* < 0.01). Moreover, more patients in the Dara-VMP group than in the VMP group remained MRD negative after 6 months (16% in the Dara-VMP group vs. 5% in the VMP group; *p* < 0.01) and after 12 months (14% in the Dara-VMP group vs. 3% in the VMP group; *p* < 0.01).

At a median follow-up of 40.1 months, median (m)PFS was significantly higher in the experimental arm vs. the standard arm: 36.4 months vs. 19.3 months, respectively, with a HR of 0.42 (95% CI 0.34–0.51, *p* < 0.01). Interestingly, the PFS advantage for the Dara-VMP arm was particularly evident after the completion of the 9 VMP cycles (with a sustained response rate after 18 months of 77% vs. 60%), thus suggesting that the continuous administration of daratumumab as maintenance therapy may be more beneficial than fixed-duration therapy followed by observation. Median (m)OS was not reached (NR) in either group, although a significant benefit in OS was observed in the Dara-VMP group. The HR for death in the Dara-VMP group compared to the VMP group was 0.60 (95% CI 0.46–0.80, *p* < 0.01). The Kaplan–Meier estimate of the 36-month rate of OS was 78.0% in the Dara-VMP group vs. 67.9% in the VMP group. A trend toward a higher OS was observed in all subgroup analyses, regardless of age or cytogenetic risk, even if it was less pronounced in patients with high cytogenetic risk as compared with patients with standard cytogenetic risk. No safety concerns were observed in the experimental arm, with G1–2 respiratory tract infections as the most common toxicity during the maintenance phase. However, a slightly increased number of G3–4 infections was observed in the Dara-VMP arm (22% vs. 15%).

Following the results of the ALCYONE trial, the combination of daratumumab with VMP was approved by the Food and Drug Administration (FDA) in May 2018 and, shortly after, by the European Medicines Agency (EMA).

The MAIA trial [16] was a multicenter, randomized, open label, active-controlled, phase III study that enrolled NTE NDMM patients who were randomized 1:1 to receive Rd (standard arm) vs. Dara-Rd (experimental arm). In the Dara-Rd group, daratumumab was administered intravenously every week during cycles 1–2, every 2 weeks during cycles 3–6, and every 4 weeks from cycle 7 until progression or unacceptable toxicity. A total of 737 patients were randomized.

In the intention-to-treat population, the ORR was significantly higher in the daratumumab group than in the control group (93% vs. 82%, respectively, *p* < 0.001), as were the CR rate (50% vs. 27%, respectively, *p* < 0.001), and the ≥VGPR rate (81% vs. 55%, *p* < 0.001). Moreover, the percentage of MRD-negative patients (evaluated by NGS, threshold of 1 tumor cell per 105 white cells) was more than 3 times higher in the daratumumab group than in the control group (24.2% vs. 7.3%, *p* < 0.001). At a median follow-up of 36.4 months, the mPFS was significantly higher in the experimental arm vs. the standard arm: NR vs. 33.8 months, respectively, with an HR of 0.56 (95% CI 0.44–0.71, *p* < 0.0001) [39]. The benefit in PFS was confirmed in all subgroup analyses, except in patients with baseline hepatic impairment. AEs were comparable in both standard and experimental arms, with a slightly higher incidence of G3–4 neutropenia (50% vs. 35%) and infections (32% vs. 23%) in the Dara-Rd group.

The triplet Dara-Rd was approved by the FDA in June 2019 and by the EMA in October 2019. Recently, the PEGASUS study compared Dara-Rd to the other standard-of-care treatments for elderly NDMM patients in an indirect treatment comparison analysis. Dara-Rd was associated with an inferior risk of disease progression or death, as compared to both VRd (HR for PFS 0.68, 95% CI 0.48–0.98) and Vd (HR for PFS 0.48, 95% CI 0.33–0.69) [44]. Although this study was not a head-to-head comparison of Dara-Rd with the other regimens, it further confirmed the benefit added by daratumumab to standard Rd.

### 4.2. New Combinations under Evaluation in Clinical Trials 

#### 4.2.1. Daratumumab-Based Regimens

Many other treatment combinations, based on various mAbs, are currently under investigation. Among them, the HOVON 143 phase II trial enrolled intermediate-fit and frail NDMM patients to evaluate the efficacy and safety of a first-line treatment with the association of daratumumab, ixazomib, and low-dose dexamethasone [28]. The treatment schedule included an induction phase followed by maintenance therapy. The induction consisted of 9 cycles every 4 weeks of ixazomib, 4 mg orally administered on days 1, 8, and 15; daratumumab, 16 mg/ms intravenously administered every week during cycles 1–2, every 2 weeks during cycles 3–6, and every 4 weeks during cycles 7–9; plus dexamethasone, 20 mg every week during cycles 1–2, 10 mg every 2 weeks during cycles 3–6, and 10 mg every 4 weeks during cycles 7–9. The maintenance phase continued for a maximum of 2 years and consisted of 8-week cycles with ixazomib, 4 mg on days 1, 8, 15, 29, 36, and 43; daratumumab, 16 mg/ms on day 1; plus dexamethasone, 10 mg on day 1. A total of 46 patients (23 intermediate-fit and 23 frail) were evaluated in the interim analysis. Hematologic toxicity was mild and non-hematologic AEs were manageable, with similar rates of G3–4 infections (9% in both intermediate-fit and frail patients) and cardiac events (8% in both intermediate-fit and frail patients) representing the most frequent toxicities. No early deaths (e.g., ≤3 months) were reported in intermediate-fit patients, while the early-death rate was 12% in the frail group, mainly due to vulnerability and infections. During induction, the ORR was 87% (PR 48% and VGPR 39%) and 78% (PR 48% and VGPR 26%) in intermediate-fit and frail patients, respectively. This combination was effective in both intermediate-fit and frail patients, but the high rate of early mortality in the latter group suggests better identification and support of frail patients receiving this triplet.

#### 4.2.2. Isatuximab-Based Regimens

Isatuximab is another anti-CD38 mAb characterized by a strong anti-tumor activity via direct tumor targeting and immune cell engagement. Isatuximab has been investigated in first-line NTE MM patients. Ocio et al. presented the preliminary results from a phase Ib study (NCT02513186) of isatuximab plus VRd (Isa-VRd) or bortezomib-cyclophosphamide-dexamethasone (Isa-VCd) in this setting of patients [41,42]. Treatment included an induction and a maintenance phase. Preliminary data suggested that isatuximab plus VRd or VCd was well tolerated, with the most frequent AEs of any grade being constipation, IRRs, diarrhea, and peripheral sensory neuropathy. G3–4 AEs were reported in 46% of patients in the Isa-VRd cohort vs. 65% of patients in the Isa-VCd cohort. Updated results showed an ORR of 100% in the Isa-VRd cohort and 93.3% in the Isa-VCd cohort. No patient had progressed at a median follow-up of 21 and 37 months, respectively, thus suggesting the high efficacy of this regimen [43]. The phase III IMROZ study is currently comparing the quadruplet Isa-VRd to VRd as upfront treatment for NTE patients (NCT03319667). Another ongoing trial is comparing the quadruplet Isa-VRd to Isa-VCd (NCT02513186).

#### 4.2.3. Elotuzumab-Based Regimens

Aside from anti-CD38 mAbs, other molecules have been investigated in treatment combinations in previously untreated NTE MM patients. Among them, the anti-SLAMF7 mAb elotuzumab represents a promising option for elderly patients, due to the low rate (about 10%) of IRRs, which are usually mild (G1–2) and do not lead to treatment discontinuation. The ELOQUENT-1 trial is a randomized, open-label, phase III study evaluating the combination of elotuzumab with standard Rd (Elo-Rd) vs. Rd in this setting of patients. This study is currently ongoing, and preliminary results showed that there was no statistical improvement in terms of PFS in the Elo-Rd arm as compared with the Rd arm [45]. The safety profile of Elo-Rd was good and consistent with that previously reported with the same combination in the RR setting [35].

Elotuzumab was also evaluated in association with VRd in the randomized, phase II trial SWOG-1211 [40], which compared 8 cycles of VRd induction followed by dose-attenuated VRd maintenance until disease progression, with or without elotuzumab. No safety concerns emerged. However, at a median follow-up of 53 months, no differences were observed in terms of mPFS (Elo-VRd vs. VRd: 31 vs. 34 months, HR 0.968, *p* = 0.449) and OS (Elo-VRd vs. VRd: 68 months vs. NR, HR 1.279, *p* = 0.478).

Many other ongoing trials are currently investigating different mAbs that showed activity in various combinations for the treatment of first-line NTE MM patients. These data are expected soon [46,47,48,49].

#### 4.2.4. Pembrolizumab-Based Regimens

Pembrolizumab is a mAb of the family of checkpoint inhibitors, and it is directed against the PD-1 receptor. Preliminary phase I/II trials of pembrolizumab in combination with immunomodulatory agents showed encouraging efficacy and acceptable toxicity [50,51]. Following this evidence, the phase III KEYNOTE-185 trial comparing Rd with and without pembrolizumab in NTE MM patients was initiated. Nevertheless, this trial was prematurely terminated due to safety concerns. An interim analysis at 6 months (requested by the FDA) reported significantly higher rates of G3–4 AEs (64% vs. 45%) and toxic deaths (4% vs. 1%) in the pembrolizumab arm than in the control arm. Moreover, 23% of patients experienced G ≥ 3 immune-mediated AEs. Median PFS and OS data will not be reached, but similar ORRs (64% vs. 62%) and PFS rates at 6 months (82% vs. 85%) were observed in the two arms [52]. According to these results, as well as in light of the safety and efficacy results with the available daratumumab triplets/quadruplets, further development of pembrolizumab for the treatment of elderly NDMM patients is unlikely.

## 5. Discussion

Following the results of the ALCYONE and MAIA trials, Dara-VMP and Dara-Rd became new standards of care for NTE NDMM patients. In the next years, the approval of these new combinations will likely result in a further benefit in survival to elderly MM patients. Since data from literature showed that almost 50% of elderly MM patients will undergo a second line of treatment, the importance of more efficient first-line therapy makes these results even more interesting. Besides daratumumab, isatuximab showed outstanding results in combination with bortezomib, steroids, and cyclophosphamide/lenalidomide in preliminary studies, and ongoing phase III trials might lead to new approved combinations. Nevertheless, this radical change in the treatment scenario leaves unanswered questions.

Although the addition of daratumumab improved efficacy of both VMP and Rd, choosing between Dara-VMP and Dara-Rd may be challenging because no head-to-head comparison between these two combinations is currently available. Both combinations reduced the risk of disease progression or death, as compared to standard arms in subgroups of patients with impaired renal function (PFS: HR 0.36 vs. 0.60 in Dara-VMP vs. Dara-Rd, respectively), ISS stage III (HR 0.53 vs. 0.72, respectively), high-risk cytogenetics (HR 0.78 vs. 0.85, respectively), and in ≥75-year-old patients. Concerning feasibility, less than 10% of patients discontinued treatment due to AEs with both Dara-VMP and Dara-Rd, and no safety concerns arose during the trials [15,16]. No data from clinical practice about these daratumumab-based combinations administered in unselected real-life patients are yet available. Consequently, to date both regimens are valid options, and the choice of one regimen over another may be guided by comorbidities and patient preference. A trial exploring a head-to-head comparison between VMP vs. Rd in a real-world population is being conducted in Italy (NCT03829371), and its results can help identify subpopulations of patients that benefit from one backbone regimen over another.

The manageability of mAbs in terms of safety changed treatment approach for elderly patients, introducing continuous therapy at first line. Indeed, aside from Rd, traditional regimens such as VMP, VRd, or melphalan-prednisone-thalidomide were administered for a fixed duration, due to the risk of long-term toxicity. Daratumumab maintenance after 9 VMP cycles was demonstrated to be safe and to further increase the survival benefit, as compared to observation [38]. In the MAIA trial, the whole triplet Dara-Rd is administered until progression. The effect of continuing daratumumab while reducing lenalidomide dose or discontinuing dexamethasone beyond the first cycles has never been evaluated. Preliminary data of a phase III trial reported that, in patients receiving Rd, reducing lenalidomide dose and discontinuing dexamethasone after the first 9 cycles did not affect efficacy but limited toxicity, as compared to full-dose Rd [53]. Future trials may evaluate this approach, which appears particularly appealing for elderly patients.

Nevertheless, daratumumab administration until disease progression entails monthly hospital admissions for intravenous infusions. In this view, better ways to deliver daratumumab have been evaluated. Recently, rapid daratumumab infusion over 90 min (compared to standard 3 h) after the first 2 infusions was evaluated, without either significant safety concerns or increase in IRR rates [54]. Subcutaneous delivery of daratumumab (at a flat dose of 1800 mg co-formulated with recombinant human hyaluronidase PH20) showed a good safety profile in RRMM patients, and this route of administration has been included in the design of many ongoing trials [55,56]. These options could be particularly appealing for elderly patients, reducing the burden of long hospital stays and improving the quality of life [57].

Another important issue is the choice of treatment in elderly patients who are not fit enough to receive full-dose mAb-based triplets/quadruplets. Indeed, in the ALCYONE, MAIA and IMROZ trials, patients with poor performance status (Eastern Oncology Group [ECOG] performance status >2) or significant comorbidities (e.g., renal impairment with creatinine clearance <30/40 mL/min, hepatic impairment, active cardiovascular diseases) were excluded. Gentler combinations are under evaluation in frailty-tailored approaches, as described above. MAbs also seem to be effective in this subset of patients. Nevertheless, toxicity is often a concern, with infections representing one of the major causes of morbidity and mortality. Indeed, in patients receiving daratumumab or isatuximab in combination with other novel agents, the rate of G ≥ 3 infections was 25–35% [15,16,43]. Careful patient selection, dose adjustments (e.g., dexamethasone-sparing strategies) and adequate supportive care and prophylaxis should be warranted for frail patients. In NDMM patients, prophylactic levofloxacin administered for the first 3 months of therapy should be considered, since it demonstrated to reduce febrile episodes and deaths, as compared to placebo (HR 0.66, *p* = 0.001) [58].

Finally, given the continuous evolution of the MM treatment scenario, other immunotherapeutic approaches besides naked mAbs are beginning to emerge. Antibody-drug conjugates (ADCs) such as belantamab mafodotin showed impressive results in the relapse setting and a manageable safety profile, with the main toxicities being thrombocytopenia and corneal events [59]. The ongoing DREAMM-9 trial (NCT04091126) is evaluating belantamab mafodotin in combination with VRd vs. VRd in NTE NDMM patients. Bispecific antibodies (BiAbs) are also in the spotlight because of the high efficacy demonstrated in early-phase trials in RRMM patients. No data about the administration of BiAbs in elderly NDMM patients are available, but toxicity—particularly in terms of neurotoxicity, cytokine release syndrome, and infections—may be a concern for their feasibility in the treatment of elderly patients.

Future trials should be designed on specific populations according to frailty status: Now that we have access to new immunotherapeutic agents (ADCs, BiAbs) [12], in which population should we explore using them? BiAbs should be explored for the treatment of fit patients, where efficacy and the achievement of deep responses are the treatment goals, and patients are likely to have enough organ function to tolerate neurotoxicity/cytokine release syndrome and/or infections. However, BiAbs are not easily combined with other agents and, as a consequence, they might be used as consolidation therapy in patients reaching an unsatisfactory response (e.g., MRD-positive patients after initial treatment). 

ADCs (among which is belantamab mafodotin) are easier to be combined with MM backbone treatments and may be particularly interesting for the treatment of fit/intermediate-fit elderly patients, due to their high efficacy (possibly higher than anti-CD38 mAbs) coupled with a low systemic toxicity. Corneal events may lead to a deterioration of the quality of life, especially in elderly patients. Early recognition and mitigation strategies should therefore be pursued to contain these events. The long-term use of naked mAbs (especially if administered subcutaneously) demonstrated to be very safe and convenient in elderly MM patients and it could really change the outcome of frail MM patients if coupled with lower doses of backbone treatments, steroid-sparing strategies, and optimal supportive therapies.

The concerns, opportunities and possible future directions regarding the use of the aforementioned mAb classes in elderly NDMM patients are summarized in Table 2.

## Figures and Tables

**Table 1:** Data about efficacy and safety of mAb-based treatments in NTE NDMM patients.

**a. Efficacy**							
**Trial**	**N pts**	**Treatment**	**OR (%)**	**CR/VGPR (%)**	**MRD neg (%)**	**mPFS** **[95% CI] (Months)**	**mOS** **[95% CI] (Months)**
**ALCYONE** [38]	706	Dara-VMP	90.9	43/71	28	36.4	78.0
		vs.	vs.	vs.	vs.	vs.	vs.
		VMP	73.9	25/50	7	19.3	67.9
**MAIA** [16,39]	737	Dara-Rd	93	50/81	24.2	NR	NR
		vs.	vs.	vs.	vs.	vs.	vs.
		Rd	82	27/55	7.3	33.8	NR
**HOVON 143** [28]	46	Dara-Ixa-dex			-	*9-month PFS rates*:	*9-month OS rates*:
		interm.-fit	87	0/39		78 (55–90)	100
		vs.	vs.	vs.		vs.	vs.
		frail pts	78	4/26		61 (38–77)	83 (60–93)
**SWOG-1211** [40]	103	Elo-VRd	-	-	-	31	68
		vs.				vs.	vs.
		VRd				34	NR
**TCD13983** [41,42,43]	44	Isa-VRd	100	41/52	30.8	NR	-
		vs.	vs.	vs.	vs.	vs.	
		Isa-VCd	93.3	59	33.3	NR	
**b. Safety**							
**Trial**	**N pts**	**Treatment**	**Total Discontinuation (%)**	**Discontinuation due to AE (%)**	**Discontinuation due to Death (%)**	**Grade 3–4 Hematologic Toxicities: Neutropenia/Anemia (%)**	**Grade 3–4 Non-Hematologic Toxicities: Infections (%)**
**ALCYONE** [38]	706	Dara-VMP	-	7	24	40/15	22
		vs.		vs.	vs.	vs.	vs.
		VMP		9	36	39/20	15
**MAIA** [16,39]	737	Dara-Rd	39	9	23	51/14	36
		vs.	vs.	vs.	vs.	vs.	vs.
		Rd	64	18	28	35.3/21	27
**HOVON 143** [28]	46	Dara-Ixa-dex					
		Interm.-fit	30	4	0	8/-	9
		vs.	vs.	vs.	vs.	vs.	vs.
		frail pts	39	0	13	17/-	9
**SWOG-1211** [40]	103	Elo-VRd	-	-	-	-	16
		vs.					vs.
		VRd					8
**TCD13983** [41,42,43]	22	Isa-VRd	14	9	5	12.5%	27

Abbreviations: NTE, transplant ineligible; NDMM, newly diagnosed multiple myeloma; ORR, overall response rate; CR, complete response; VGPR, very good partial response; MRD, minimal residual disease; neg, negativity; mPFS, median progression-free survival; mOS, median overall survival; NR, not reached, AE, adverse event; CI, confidence interval; Dara, daratumumab; V, bortezomib; M, melphalan; P, prednisone; R, lenalidomide; d, dexamethasone; Ixa, ixazomib; Elo, elotuzumab; Isa, isatuximab; C, cyclophosphamide; interm.-fit, intermediate fit; N, number; pts, patients.

**Table 2:** Concerns, opportunities and possible future directions regarding the use of selected anti-MM mAb classes for the treatment of NTE NDMM patients.

Drug Class	Concerns in Elderly Patients	Opportunities in Elderly Patients	Possible Future Directions
Anti-CD38 mAbs	IRRs, slight increase of infection risk	Synergy with other MM backbone treatments, easy to combine	Part of standard-of-care regimens for virtually all NTE NDMM patients?
Anti-SLAMF7 mAbs	Low efficacy	Very good safety, easy to combine	Exploration of their use in new combination therapies? With other immune therapies?
Checkpoint inhibitors	immune-mediated AEs, infections	Possible synergy with IMiD-based backbones	Suboptimal as compared with available alternatives.
ADCs	Corneal events	High efficacy as single agent, feasible combination with other MM backbone treatments	Exploration of their use in combination with other backbone MM treatments?
BiAbs	CRS, neurotoxicity, infections	Very high efficacy as single agent	Consolidation therapy in fit, MRD-positive, NTE NDMM patients?

Abbreviations: NTE, transplant-ineligible; MM, multiple myeloma; NDMM, newly diagnosed MM; mAbs, monoclonal antibodies; IRRs, infusion-related reactions; AEs, adverse events; IMiDs, immunomodulatory drugs; ADCs, antibody-drug conjugates; BiAbs, bispecific antibodies; CRS, cytokine release syndrome; MRD, minimal residual disease.
